# Preoperative and Postoperative Arterial Adaptation in Patients with Acute Aortic Dissection

**DOI:** 10.3390/jcm13237362

**Published:** 2024-12-03

**Authors:** Marian Burysz, Mariusz Kowalewski, Natalia Piekuś-Słomka, Jerzy Walocha, Jarosław Zawiliński, Radoslaw Litwinowicz, Jakub Batko

**Affiliations:** 1 Department of Cardiac Surgery, Regional Specialist Hospital, 86-300 Grudziądz, Poland; 2 CAROL—Cardiothoracic Anatomy Research Operative Lab, Department of Cardiovascular Surgery and Transplantology, Institute of Cardiology, Jagiellonian University Medical College, 31-008 Kraków, Poland; 3 Thoracic Research Centre, Collegium Medicum, Nicolaus Copernicus University, Innovative Medical Forum, 85-067 Bydgoszcz, Poland; 4 Faculty of Medicine, Bydgoszcz University of Science and Technology, 85-796 Bydgoszcz, Poland; 5 Department of Cardiac Surgery, Central Clinical Hospital of the Ministry of Interior, Centre of Postgraduate Medical Education, 02-507 Warsaw, Poland; 6 Cardio-Thoracic Surgery Department, Heart and Vascular Centre, Maastricht University Medical Centre, 6229 HX Maastricht, The Netherlands; 7 Department of Inorganic and Analytical Chemistry, Faculty of Pharmacy, Nicolaus Copernicus University, 85-089 Torun, Poland; natalia.piekus@cm.umk.pl; 8 Department of Anatomy, Jagiellonian University Medical College, 31-008 Kraków, Poland

**Keywords:** spinal cord ischemia, aortic aneurysm, frozen elephant trunk

## Abstract

**Background:** Spinal cord ischemia is one of the most serious complications after an aortic repair. To date, there is no evidence for arterial changes during an aortic dissection or for the observation of such arteries after an aortic repair. The aim of this study was to compare spinal-cord-supplying arteries in patients with an acute aortic dissection, preoperatively and postoperatively, with patients without an acute aortic dissection. **Methods:** Preoperative and postoperative contrast-enhanced computed tomography scans of 25 patients who had undergone the frozen elephant trunk procedure to treat an aortic dissection and 25 patients who qualified for a transcatheter aortic valve replacement without an acute aortic dissection and atherosclerosis of the analyzed vessels, treated as a control group, were reconstructed and retrospectively analyzed with the detailed medical data of the patients. The aortic branches with the ability to supply blood to the spinal cord as described in the literature were further analyzed. **Results:** The preoperative arterial diameters of the left internal thoracic artery, the left musculophrenic artery, and the left and right supreme intercostal arteries were significantly larger compared to the postoperative measurements. In addition, the preoperative measurements of the diameters of the left vertebral artery, right internal thoracic artery, left lateral thoracic artery, and left common iliac artery were significantly larger than in the control group. **Conclusions:** The internal thoracic arteries and supreme intercostal arteries may play a crucial role in providing additional blood supply to the spinal cord.

## 1. Introduction

Spinal cord ischemia is one of the most serious complications of an aortic repair, observed in approximately 1/600 patients with an unruptured aortic aneurysm to 1/130 patients with an acute rupture, regardless of the repair technique [1,2]. The intraoperative development of spinal cord ischemia may be caused by inadequate blood flow, a clot, or an intraoperative spasm of the radicular arteries [3]. Late-onset spinal cord ischemia, which can be irreversible, should also be mentioned [4,5].

The blood supply to the spinal cord is mainly provided by the radicular arteries, originating from spinal branches of arteries adjacent to the vertebral column, with additional branches emanating from the surrounding blood vessels in each spinal cord segment, forming the vasocorona [6,7]. The best-known radicular artery is the artery of Adamkiewicz, named after Dr. Albert Adamkiewicz, who developed the first complete description of the blood supply to the spinal cord. He theorized that it is crucial for the blood supply to the spinal cord in the lower part of the thoracic segment, lumbar segment, and conus medullaris. Based on his observations, damage to the Adamkiewicz artery is recognized as one of the main risk factors for spinal cord ischemia during an aortic repair [8]. However, the studies investigating the efficacy of reconstruction in preventing spinal cord ischemia have not shown any significant improvement in patients undergoing such a procedure [9].

The most recent theory describing the blood supply to the spinal cord—the collateral network theory—was developed by Kadyi in the XIXth century and was rediscovered by Dr. Griepp and Dr. Maliszewski in the 1990s [10,11,12]. It describes the importance of dense anastomoses between multiple supplying arteries as crucial for maintaining blood flow in the spinal cord as opposed to a single main artery. This theory was further supported by experimental studies in animal models and clinical observations of patients undergoing an aortic repair [13,14]. Further studies have emphasized the importance of damage to the subclavian and iliac arteries in the development of ischemic complications as potential sources of blood supply to the collateral network of the spinal cord [15,16].

To date, there is a lack of evidence regarding the importance of other types of arterial damage associated with the spinal cord supply, the incidence of spinal cord ischemia, arterial changes during an aortic dissection, and the observation of these arteries after an aortic repair.

The aim of this study was to compare spinal-cord-supplying (SCS) arteries in patients with an acute aortic dissection, preoperatively and postoperatively, with patients without an acute aortic dissection.

## 2. Materials and Methods

### 2.1. Study Population

Preoperative and postoperative [10 (7–14) days] contrast-enhanced computed tomography (CT) scans of the thorax and abdomen of 25 (aged 60 (53–67), 40.0% female) patients who underwent the frozen elephant trunk procedure for the treatment of an acute aortic dissection involving the aortic arch, which was described in detail in our previous study [17], and 25 (aged 76 (71–80), 40.0% female) patients who qualified for a transcatheter aortic valve replacement without acute aortic dissection, dilatation of the aorta, and atherosclerosis of the analyzed vessels, treated as a control group, were reconstructed and retrospectively analyzed with the detailed medical data of the patients.

### 2.2. Imaging Characteristics and Analysis

The CT scans were performed with a 64-row dual-source scanner (Briliance 64, Philips, 595 Miner Rd, Cleveland OH, USA). The imaging parameters for CT were as follows: a 120 kV tube voltage and a 340–400 mA effective tube current. The slice gradation was 0.5 mm and the slice thickness was 1.0 mm. The images were reconstructed with an image matrix of 512 × 512 pixels. An amount of 1 mL/kg of an iodine contrast agent was used per patient. All the CT scans were analyzed on a special workstation (Dell, Round Rock, TX, USA). The raw data and visualizations were further analyzed morphologically and morphometrically using virtual calipers and the Mimics Innovation Suite 24 software (Materialize, Plymouth, MI, USA).

### 2.3. Visualizations and Measurements

The entire aorta and its branches were reconstructed in each study group. The aortic branches were categorized into an SCS group, based on the possibilities of blood supply to the spinal cord as described in the literature [18].

**Figure 1:**
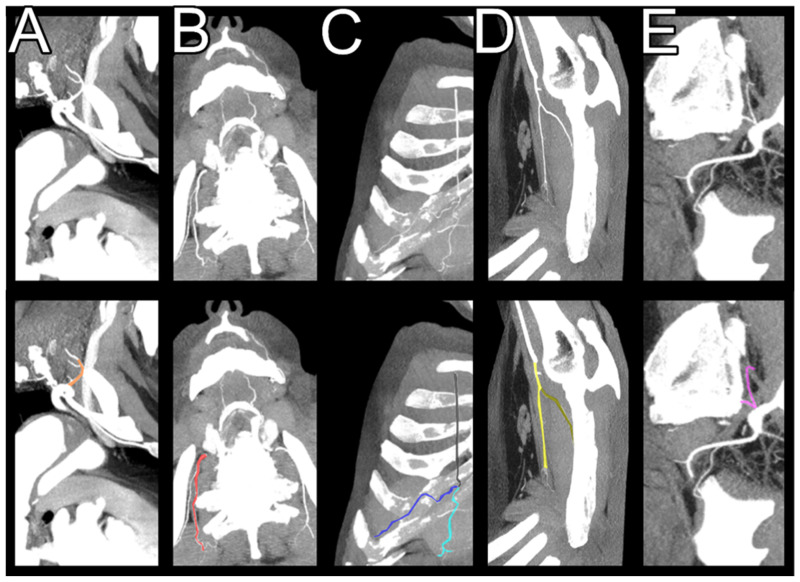
Spinal-cord-supplying arteries. Multiplanar reconstruction with maximum intensity projection. (**A**)—Costocervical trunk, marked with orange. (**B**)—Supreme intercostal artery, marked with red. (**C**)—Internal thoracic artery with its branches. Internal thoracic artery is marked with black, superior epigastric artery is marked with light blue, and musculophrenic artery is marked with dark blue. (**D**)—Lateral thoracic artery. Lateral thoracic artery is marked with yellow and thoracodorsal artery is marked with dark yellow. (**E**)—Iliolumbar artery, marked with purple.

The SCS arteries included arteries whose branches can supply the spinal cord: the brachiocephalic trunk, the left common carotid artery, the left subclavian artery, the common iliac arteries, the vertebral arteries, the superior intercostal arteries, the internal and lateral thoracic arteries, the superior epigastric arteries, the musculophrenic arteries, the superior epigastric arteries, the iliolumbar arteries, and the costocervical trunks (Figure 1).

The diameter of each artery, 2 mm from the origin, was measured on the preoperative, postoperative, and control group CT scans. The brachiocephalic trunk, left common carotid artery, and left subclavian artery were measured 2 mm above the anastomosis with the graft postoperatively.

### 2.4. Statistical Analyses

The collected data were tested for normality using the Shapiro–Wilk test, and were tabulated and presented as the median with the first and third quartiles [median (Q1–Q3)]. The U-Mann–Whitney test was used to compare patients with an AAA, before and after surgery, with the control group. The Wilcoxon test was used to analyze the differences between the preoperative and postoperative diameters of each vessel. The data were analyzed using IBM SPSS Statistics 29.0 (Predictive Solutions, Pittsburgh, PA, USA).

### 2.5. Ethical Statement

The authors of this study take full responsibility for the accuracy and integrity of the research and have taken appropriate measures to ensure that all concerns regarding the work have been addressed. The study was conducted in accordance with the ethical principles of the Declaration of Helsinki (as revised in 2013). The study was approved by the Jagiellonian University Ethics Committee (No.: 1072.6120.48.2022), and the need for individual informed consent was waived for this retrospective analysis.

## 3. Results

### 3.1. Characteristics of the Patients

With the exception of age (60 (53–67) vs. 76 (71–80), *p* < 0.001), there were no significant differences between the study and control groups with regard to comorbidities at baseline. The detailed characteristics of the patients are described in Table 1.

### 3.2. Comparison of the Preoperative, Postoperative, and Control Group SCS Arteries

Compared to the postoperative measurements, differences were found for the preoperative arterial diameters of the left internal thoracic artery (significantly larger, *p* = 0.04), the left musculophrenic artery (significantly larger, *p* = 0.04), and the left and right supreme intercostal arteries (significantly larger, *p* < 0.001). This observation was maintained for each of the above arteries compared to the control group. In addition, the preoperative measurements of the diameters of the left vertebral artery, right internal thoracic artery, left lateral thoracic artery, and left common iliac artery were significantly larger than in the control group. Postoperatively, the diameters of the left and right vertebral arteries and the left and right common iliac arteries were significantly larger than in the control group. The detailed measurements are listed in Table 2.

### 3.3. SCS Arteries in a Patient with Spinal Cord Ischemia

A 59-year-old patient presented to our cardiac surgery department with a diagnosis of an AAD of the ascending aorta and its arch, which was confirmed by CT. Prior to surgery, the patient had a sudden cardiac arrest that returned to spontaneous circulation after resuscitation. The patient’s BMI was 34.0 and their Euroscore was 24.6. The patient had a history of smoking and hypertension. The laboratory results revealed decreased hemoglobin (11.7 g/dL), red blood cells (3.8 mln/uL), and hematocrit (35.2%) and increased C—reactive protein (90.6mg/L), aspartate aminotransferase (184 U/L), and glutamate pyruvate transferase (164 U/L). A size 32/40/150 prosthesis was implanted. Detailed information on the procedure can be found in our previous study [17]. Postoperatively, the patient developed paraplegia. A postoperative CT was performed, which confirmed the correct localization of the prosthesis. The patient died 8 months after surgery due to sepsis. In the retrospective analysis, the diameters of the left and right superior intercostal artery were 1.2 mm and 1.3 mm preoperatively, respectively, and no changes were found postoperatively. The diameters of the left and right internal mammary arteries were 2.6 mm and 2.8 mm, respectively, and 2.4 mm and 2.9 mm, respectively, postoperatively. The left and right vertebral arteries were 4.5 mm and 4.3 mm preoperatively and 5 mm and 4.6 mm postoperatively.

## 4. Discussion

An aortic aneurysm is a critical condition associated with local and systemic adaptive mechanisms that are not yet fully understood. Our study provides a detailed preoperative and postoperative analysis of the aortic branches in patients with this condition. Previous studies have provided clinical evidence of the clinical importance of the left subclavian artery in patients undergoing an aortic aneurysm repair [15,16]. Although there were no statistically significant differences between the diameter of the left subclavian artery in patients with an aortic aneurysm and the control group, such differences were observed in its branches, including the left internal thoracic artery with its branch—the musculophrenic artery, the left vertebral artery, the left supreme intercostal artery—of the costocervical trunk, and the left lateral thoracic artery, one of the branches of the axillary artery. The above-mentioned left internal thoracic artery with its branch showed significant differences pre- and postoperatively. This observation was not maintained for the arteries arising from the right subclavian artery. It should be noted that such an adaptation was observed bilaterally for the supreme intercostal arteries. In patients with developed ischemic complications, we did not observe a preoperative increase in the diameter of the internal mammary arteries or the superior intercostal arteries. Postoperatively, their diameter remained similar. The vertebral arteries of the aforementioned patient were significantly enlarged preoperatively, and a further increase was observed after the procedure. This observation should be taken with caution, as this was the only case of spinal cord ischemia observed in our center after the frozen elephant trunk procedure.

As far as the blood supply to the spinal cord is concerned, the main source is the anterior and posterior radicular arteries, which are supplemented by several other arteries in each spinal cord segment [18]. In the cervical segment, this supply is observed from the vertebral artery, the carotid artery, and the inferior thyroid artery [18]. In the thoracic and lumbar spine segments, the vertebral arteries are supplied by the superior intercostal arteries, the posterior intercostal arteries, and the lumbar and iliolumbar arteries [18].

In addition, the internal thoracic artery is connected to the intercostal arteries via an anastomosis of its branch—the musculophrenic artery—which can ensure blood flow in the event of an interruption of blood flow in the intercostal arteries due to an aortic aneurysm (Figure 1). According to our study, their diameter increases significantly preoperatively and does not differ significantly after an aortic repair from the arteries observed in patients without an aortic rupture. This observation was more pronounced in the left internal thoracic artery than on the right side.

The supreme intercostal artery, which originates from the costocervical trunk, one of the branches of the subclavian artery, supplies blood to the first and second posterior intercostal arteries and, via its branches, directly to the collateral network of the spinal cord [18]. However, the dense network of horizontal and vertical anastomoses between the segmental arteries allows blood to flow through them to more distal intercostal arteries in patients with an aortic aneurysm.

The above observations may lead to crucial insights related to the previously well-documented shock-induced arterial adaptation, particularly the centralization of circulation. Arteries with a significantly enlarged lumen during an aortic aneurysm may play an additional role in protecting the central nervous system, particularly the spinal cord. After an aortic repair, this additional blood supply may no longer be necessary, leading to a postoperative decrease in the diameter of the SCS arteries. This mechanism could be related to the clinically observed late spinal cord ischemic complications in patients without the proper postoperative recruitment of the paraspinal anastomoses. As demonstrated in our study, these arteries can be examined with CT, which is typically performed in patients with an aortic aneurysm [19,20,21,22].

Future studies should analyze whether such phenomena can also be observed in patients with a chronic aortic dissection and what impact this has on patient survival and complication rates. In addition, patients who have undergone an aortic repair with techniques other than the frozen elephant trunk should be analyzed to determine whether such changes can be observed in patients treated with other repair techniques.

### Limitations

Several limitations should be mentioned. This was a retrospective analysis at a single center with 25 patients. Patients with an aortic dissection at different levels of the aorta were included in this study. The statistical significance of the correlation between the arterial adjustment and clinical complications could not be assessed because only one patient with spinal cord ischemia was observed in our study population.

## 5. Conclusions

The diameter of the arteries supplying the spinal cord increases in patients with an acute aortic dissection and decreases again after the aortic repair. The internal thoracic arteries and the supreme intercostal arteries could play a decisive role in the additional blood supply to the spinal cord.

## Figures and Tables

**Table 1:** Patient characteristics.

		AAD (*n* = 25)	TAVI (*n* = 25)	*p*
Age (years)		60 (53–67)	76 (71–80)	<0.001
Sex		15 (60.0%)	15 (60.0%)	1.000
Body mass index (kg/m^2^)		28.7 (24.1–30.8)	27.4 (25.2–29.8)	0.87
Diabetes mellitus type 2		3 (12.0%)	6 (24.0%)	0.27
Hypertension		23 (92.0%)	23 (92.0%)	1.00
Atrial fibrillation		0 (0%)	0 (0%)	1.00
Chronic heart failure		1 (4.0%)	4 (16.0%)	0.16
Smoker	Active	17 (68.0%)	11 (44.0%)	0.20
	Previous	7 (28.0%)	11 (44.0%)	
Tamponade		5 (20.0%)	1 (4.0%)	0.08
Ejection fraction (%)		40 (40–48)	48 (40–55)	0.07
Parts of aorta involved				
Ascending		5 (20.0%)		
Ascending + arch		12 (48.0%)		
Ascending + arch + thoracic + abdominal		3 (12.0%)		
Thoracic + abdominal		5 (20.0%)		
Surgery time (min)		320 (290–450)		
Cardiopulmonary bypass time (min)		200 (158–280)		
Aortic cross-clamp time (min)		128 (110–162)		
Distal hypothermic circulatory arrest time (min)		35 (30–40)		
Prosthesis length 100		12 (48%)		
Prosthesis length 150		13 (52%)		
Blood cell transfusion		25 (100%)		
30-day mortality		1 (4%)		
Hospitalization time		20 (15-26)		

**Table 2:** Comparison of the arteries supplying the spinal cord preoperatively, postoperatively, and in the control group. AAA—acute aortic aneurysm, TAVI—transcatheter aortic valve implantation, *p* *—*p*-value for preoperative versus postoperative AAA comparison, *p* ^+^—*p*-value for preoperative versus TAVI comparison; *p* ^#^—*p*-value for postoperative versus TAVI comparison.

	Preoperative AAA	Postoperative AAA	TAVI	*p* *	*p* ^+^	*p* ^#^
Brachiocephalic trunk diameter	12.6 (10.4–14)	11 (10.2–12.1)	11.9 (9–14.4)	0.56	0.89	0.77
Left common carotid artery diameter	8.9 (8–9.5)	9 (7.8–9.5)	7.8 (7.0–9.1)	0.92	0.44	0.32
Left vertebral artery diameter	4.5 (3.9–5.2)	5.3 (4.4–6.0)	3.1 (2.4–3.6)	0.69	0.04	0.02
Right vertebral artery diameter	4.0 (3.2–4.5)	4.3 (3.5–5.1)	3.2 (2.6–3.9)	0.75	0.12	0.03
Left subclavian artery diameter	11.3 (10–11.6)	10 (9.1–10.1)	11.1 (9.9–12.2)	0.83	0.96	0.74
Left internal thoracic artery diameter	3.9 (3.3–4.8)	3 (2.2–3.5)	2.3 (2–2.5)	0.04	<0.001	0.55
Right internal thoracic artery diameter	3.0 (2.5–3.4)	2.4 (2.0–2.9)	2.2 (1.9–2.5)	0.07	0.02	0.9
Left superior epigastric artery diameter	1.4 (1.0–2.1)	1.6 (1.3–2.2)	1.3 (1.1–1.8)	0.82	1	0.64
Right superior epigastric artery diameter	1.5 (1.4–1.8)	1.4 (1.1–1.9)	1.3 (1.1–1.6)	0.91	0.89	0.95
Left musculophrenic artery diameter	2.4 (1.9–3.1)	1.6 (1.3–2.2)	1.4 (0.9–1.7)	0.04	0.03	0.83
Right musculophrenic artery diameter	1.8 (1.4–2.1)	1.4 (1.1–1.9)	1.2 (0.8–1.5)	0.69	0.12	0.74
Left costocervical trunk diameter	3.4 (2.4–3.9)	3.1 (2.3–3.8)	3.2 (2.5–3.8)	0.93	0.71	0.99
Right costocervical trunk diameter	3.2 (2.5–3.9)	3.0 (2.3–3.7)	3.4 (2.9–4.1)	0.84	0.66	0.57
Left supreme intercostal artery diameter	2.8 (2.3–3.7)	1.5 (1.3–2.1)	1.1 (0.8–1.3)	<0.001	<0.001	0.78
Right supreme intercostal artery diameter	2.3 (1.7–2.9)	1.2 (1.0–1.5)	1 (0.8–1.3)	<0.001	<0.001	0.99
Left lateral thoracic artery diameter	2.7 (2.1–3.2)	2.4 (1.9–2.8)	1.8 (1.6–2.3)	0.53	0.04	0.49
Right lateral thoracic artery diameter	2.4 (1.9–2.7)	2.5 (1.9–2.9)	2.1 (1.7–2.5)	0.93	0.76	0.54
Left common iliac artery diameter	13.5 (11.5–17.1)	14.9 (10.8–17.1)	10.4 (8.9–12.1)	0.61	0.04	0.01
Right common iliac artery diameter	12.4 (11.8–14)	14 (11.4–14.7)	10.9 (9.4–13.0)	0.21	0.67	0.03
Left iliolumbar artery diameter	4.1 (3.6–4.4)	3.9 (3.6–4.2)	3.5 (2.9–4.1)	0.89	0.56	0.77
Right iliolumbar artery diameter	3.8 (3.3–4.2)	4.0 (3.5–4.3)	3.5 (3.1–3.8)	0.82	0.76	0.34

## Data Availability

The data are available from the corresponding author upon reasonable request.
